# The role of endosymbionts in the evolution of haploid-male genetic systems in scale insects (Coccoidea)

**DOI:** 10.1002/ece3.222

**Published:** 2012-05

**Authors:** Laura Ross, David M Shuker, Benjamin B Normark, Ido Pen

**Affiliations:** 1 Department of Zoology, University of OxfordSouth Parks Road, Oxford, OX1 3PS, United Kingdom; 2 Department of Plant Soil and Insect Sciences and Graduate Program in Organismic and Evolutionary Biology, University of Massachusetts270 Stockbridge Road, Amherst, Massachusetts 01003; 3 School of Biology, University of St Andrews, Harold Mitchell BuildingSt Andrews, Fife, KY16 9TH, United Kingdom; 4 Theoretical Biology Group, Centre for Ecological and Evolutionary Studies, University of GroningenKerklaan 30, 9750 AA Haren, The Netherlands

**Keywords:** Endosymbiosis, genomic conflict, haplodipoidy, parthenogenesis, paternal genome elimination, scale insects

## Abstract

There is an extraordinary diversity in genetic systems across species, but this variation remains poorly understood. In part, this is because the mechanisms responsible for transitions between systems are often unknown. A recent hypothesis has suggested that conflict between hosts and endosymbiotic microorganisms over transmission could drive the transition from diplodiploidy to systems with male haploidy (haplodiploidy, including arrhenotoky and paternal genome elimination [PGE]). Here, we present the first formal test of this idea with a comparative analysis across scale insects (Hemiptera: Coccoidea). Scale insects are renowned for their large variation in genetic systems, and multiple transitions between diplodiploidy and haplodiploidy have taken place within this group. Additionally, most species rely on endosymbiotic microorganisms to provide them with essential nutrients lacking in their diet. We show that species harboring endosymbionts are indeed more likely to have a genetic system with male haploidy, which supports the hypothesis that endosymbionts might have played a role in the transition to haplodiploidy. We also extend our analysis to consider the relationship between endosymbiont presence and transitions to parthenogenesis. Although in scale insects there is no such overall association, species harboring eukaryote endosymbionts were more likely to be parthenogenetic than those with bacterial symbionts. These results support the idea that intergenomic conflict can drive the evolution of novel genetic systems and affect host reproduction.

## Introduction

There is an extraordinary diversity in genetic systems across species, which includes variation in ploidy level, presence or absence of sexual reproduction, and difference in sex determination mechanisms (White 1973; Norton et al. 1993; Normark 2003). However, this variation is not spread equally across the tree of life—within some higher taxa there is no variation at all while in others variation exists between closely related species or indeed within a single species. One group that stands out in terms of the extraordinary diversity of its genetic systems is the scale insects (Nur 1980; Ross et al. 2010). Scale insects comprise one superfamily within the order Hemiptera, yet there is almost as much variation in genetic systems within scale insects (see Table 1), as there is across insects as a whole (Normark 2003). Recently, ideas that intergenomic conflict can shape patterns of genetic system evolution have come to prominence (Bull 1983; Hamilton 1993; Werren and Beukeboom 1998; Burt and Trivers 2006; Uller et al. 2007), including in scale insects (Brown 1964; Normark 2004, 2006; Shuker et al. 2009; Ross et al. 2010).

**Table 1:** The genetic systems observed in scale insects (Nur 1980; Normark 2003; Ross et al. 2010).

Genetic system	Description
*Sexual systems*	
Diplodiploidy (XX-XO)	Both sexes develop from fertilized eggs and are diploid. Females are XX, males XO.
Diplodiploidy (2N-2N)	Both sexes develop from fertilized eggs and are diploid. No sex chromosomes have been observed.
Arrhenotoky	Females develop from fertilized eggs and are diploid, males develop from unfertilized eggs and are haploid.
Hermaphroditism	Diploid hermaphroditic individuals have a diploid female reproductive system producing oocytes and haploid testis cells producing sperm.
Germline paternal genome elimination (lecanoid, Comstockiella)	Both sexes develop from fertilized eggs and are diploid but in males paternal genes are deactivated during early development and subsequently not transmitted.
Embryonic paternal genome elimination (Diaspidid)	Both sexes develop from fertilized eggs and are diploid but in males paternal genes are lost during early development rendering males haploid.
Diploid arrhenotoky	Females develop from fertilized eggs and are diploid, males develop from unfertilized eggs, become diploid due to fusion of the haploid cleavage nuclei, but have haploid gene expression as one of the two-genome sets is deactivated.
*Asexual systems*	
Deuterotoky	Reproduction can be both sexual and asexual and both males and females can develop from either fertilized or unfertilized eggs. Individuals that develop from unfertilized eggs restore diploidy by fusion of the first haploid cleavage nuclei.
Automictic thelytoky	Females develop from unfertilized eggs, males are absent. Meiosis is normal and diploidy is restored either by the fusion one polar body with the pronucleus, or by the fusion of the first haploid cleavage nuclei.
Apomictic thelytoky	Females develop from unfertilized eggs, males are absent. Meiosis does not take place.

In order to understand the variation in genetic systems, we need to understand the transitions between the different systems. In scale insects it has generally been assumed that diplodiploidy with a genetic sex determination system (in this case XX-XO) is the ancestral genetic system (Nur 1980). This system is found in most of the families within the relatively species-poor paraphyletic assemblage traditionally referred to as the Archaeococcoidea. However, the majority of scale insect species have a remarkable genetic system called paternal genome elimination (PGE). In this system both sexes develop from fertilized eggs and are diploid. However, in males the chromosomes inherited from the father are deactivated during early development and subsequently lost from the germline during spermatogenesis (Schrader 1921; Brown and Nelson-Rees 1961). PGE is a synapomorphy of a major clade of scale insects, informally termed Neococcoidea. It has previously been suggested that since this remarkable system shows interesting similarities with arrhenotoky (where females develop from fertilized and males from unfertilized eggs), it might constitute an intermediate stage between diplodiploidy and arrhenotoky (Schrader and Hughes-Schrader 1931; Bull 1979, 1983). There is some evidence for this in mites (Cruickshank and Thomas 1999), but in scale insects extant PGE and arrhenotokous clades have clearly evolved independently from diplodiploidy (Cook et al. 2002). Although diplodiploidy has been assumed to be ancestral, in a few taxa diplodiploidy seems to be a derived feature resulting from a reversion from PGE to diplodiploidy (Nur 1980). These taxa, which generally lack sex chromosomes, are of particular interest when trying to understand the evolution of the variety of genetic systems in scale insects (Herrick and Seger 1999; Ross et al. 2010). Another important genetic system found in scale insects is hermaphroditism, a system found in no other insects (Hughes-Schrader 1925; Royer 1975; Normark 2003). Apart from a variety of sexual reproductive systems, asexual reproduction is also common in scale insects found in members of both the Archaeococcoidea and in the Neococcoidea. Again there is a lot of variation in the form of asexuality, with up to six different systems described (Table 1; Nur 1971; Ross et al. 2010).

Several hypotheses have been brought forward to explain the transition between genetic systems and the resulting diversity in scale insects (as reviewed by Ross et al. 2010). Although some of these hypotheses are plausible none of them have yet been formally tested and the support for each of them is generally weak (Normark 2004). One recent hypothesis focuses on the transition from diplodiploidy to systems with haplodiploidy (arrhenotoky and PGE) and considers endosymbiotic bacteria the key driver of this transition through conflicts between hosts and endosymbionts over transmission (Normark 2004). This leads to the expectation that species that have endosymbionts are more likely to have male-haploid genetic systems compared to those that do not contain endosymbionts. Here, we attempt to test this hypothesis using data from scale insects, but before we detail our methods we will first briefly review the presence and significance of endosymbionts in scale insects and describe Normark's hypothesis in more detail.

Scale insects, like many Hemiptera, feed almost exclusively on phloem of their host plant. This constitutes a problem, as phloem is very rich in sugars but poor in other nutrients, most notably in essential amino acids. In order to compensate for the imbalance in their diet, many phloem-feeding insects have engaged in a symbiotic relationship with microorganisms (Buchner 1965; Moran and Telang 1998). It has even been suggested that this evolutionary invention has allowed them to colonize a niche that would have otherwise been out of reach and has allowed the rapid diversification of phloem feeders (Gullan and Kosztarab 1997). Most scale insect species have an obligate relationship with one or several microorganisms, which live inside the host cells (Buchner 1965; Tremblay 1989, 1997). The relationship between host and endosymbiont is often close, and several endosymbiont taxa have been found to have phylogenies that parallel those of their hosts, indicating strict vertical transmission (Baumann and Baumann 2005; Downie and Gullan 2005; Gruwell et al. 2007). Scale insect endosymbionts are transmitted through the female line and a variety of mechanisms have evolved to ensure successful transmission of symbionts from a mother to offspring (Buchner 1965; Tremblay 1989). Another feature that indicates the close association between host and endosymbiont is that in many species endosymbionts are kept in specialized cells (bacteriocytes) or even in a specialized organ (the bacteriome: Buchner 1965).

Although obligate endosymbiosis is found across many Hemiptera, in scale insects the absence of endosymbionts is relatively common (at least compared to aphids and whiteflies: Buchner 1965; Tremblay 1989). There are several possible explanations for this. In a number of cases, changes in diet or feeding behaviour are associated with the loss of endo-symbionts. These include species that have switched to feeding on parenchyma tissues (Gullan and Kosztarab 1997), species that form galls (L. G. Cook, unpubl. data), and species that are obligately associated with ants (three mealybugs of the genus *Hippeococcus*) (Buchner 1965). Additionally, in the Stictococcidae, males are fed by mothers via a placenta-like structure, do not feed independently, and lack endosymbionts during all life stages, though stictococcid females do harbor endosymbionts. Within taxa that posses endosymbionts there is also variation in the type of endosymbiont (Buchner 1965; Tremblay 1989; Gruwell et al. 2004). The endosymbionts of most species are bacteria, but in several species the endosymbionts are unicellular fungi.

Normark's (2004) suggestion that the endosymbionts of scale insects might have played a role in the evolution of the observed variation in their genetic systems is based on the fact that endosymbionts are vertically transmitted, but only through the female line. This creates conflict between host and endosymbiont, as males constitute an evolutionary dead end for the symbionts. Endosymbionts are therefore selected to try to manipulate their host's reproduction toward producing more female offspring. Several endo-symbiotic bacteria have been found to manipulate host reproduction, most notably by inducing asexual reproduction, which removes the need for males altogether, thereby resolving the conflict between host and endosymbiont (Hurst et al. 1990; Stouthamer et al. 1990). Instead of manipulating host reproduction directly, another way for endosymbionts to increase their inclusive fitness is by killing their host when they find themselves in a male. This might benefit the bacteria if there is competition between host siblings and the resources that become available through the death of a male host can be utilized by its sisters, which carry bacteria that are related to those in killed males (Hurst 1991). Male-killing phenotypes have indeed been observed in several endosymbionts (Hurst 1991). Normark's (2004) hypothesis takes advantage of the fact that the life history of many species with haplodiploidy and PGE leads to strong and prolonged interactions between kin and that most of them contain endosymbiotic bacteria. Under these conditions male-killing may evolve, for instance with the endosymbionts destroying or deactivating incoming male-determining sperm. This would haploidize male offspring and generally kill them. As a result, there would be strong selection for haploid viability of males, with any mutation responsible spreading rapidly as haploid males will always pass on this mutation (haploid transmission advantage).

One problem with Normark's hypothesis is that male-killing phenotypes have only been observed for reproductive parasites that do not provide their hosts with any benefits, while the endosymbionts present in many haplodiploid and PGE species are obligate mutualists. Furthermore, while several additions to Normark's original model have since been published, confirming the plausibility of Normark's original hypothesis (Engelstadter and Hurst 2006; Ubeda and Normark 2006; Kuijper and Pen 2010), these studies also point out that the scenario is more likely when the transmission efficiency is high, which would be expected to be the case for mutualistic endosymbionts but not necessarily for reproductive parasites.

Although Normark's idea has received theoretical attention, no formal attempts have yet been made to try to test this hypothesis. Under Normark's hypothesis we would expect that species that have endosymbionts are more likely to have male-haploid genetic systems compared to those that do not contain endosymbionts. Here, we present results from a comparative analysis based on data from 582 scale insect species in 27 families. We first test if there is a relationship between the presence or absence of bacteria and their genetic system (diplodiploidy vs. male-haploid systems). Then we extend our treatment of the evolutionary significance of the association between endosymbionts and genetic system by considering a possible relationship between endosymbiont presence and asexual reproduction, including in this a role of endosymbiont identity (bacterial vs. eukaryote). Finally, we consider the importance of the intimacy of the relationship between host and the endosymbiont (i.e., specialized cells or tissues for the symbiont), as differences in how the host and endosymbiont interface inside the host may predict the extent to which endosymbionts can manipulate the host.

## Methods

### Data collection

**Figure 1:**
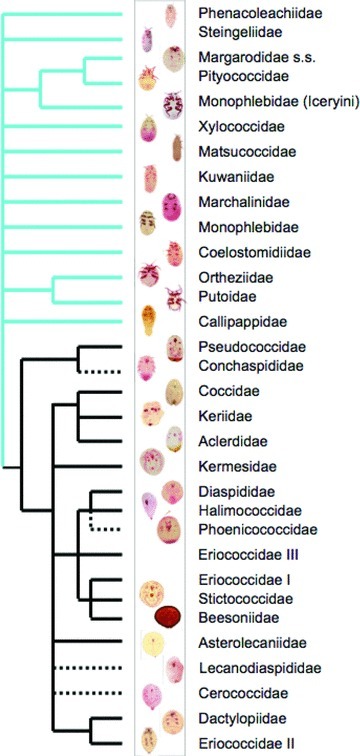
Schematic representation of the phylogenetic relationships between the Coccoid families included in our analysis. The solid lines are based on published phylogenetic studies (Cook et al. 2002; Cook and Gullan 2004; Gullan and Cook 2007; Andersen 2009) (B. B. Normark, unpubl. data), while the dashed lines show the hypothetical relationships of families for which no published sequence data are available, based on a recent review of their taxonomic status (Gullan and Cook 2007). Turquoise lines show the relationships between Archaeococcoids, while the black lines show the relationships between the Neococcoids. The pictures show slide-mounted specimens of representative species of each family and were taken from http://www.sel.barc.usda.gov/ScaleKeys/ScaleFamilies/key/Scale%20Families/Media/Html/ScaleFamilies/ReferenceFset.html.

The data used for this analysis were collected from the literature between April 2007 and January 2010. We used a variety of sources. The main source for information on the genetic systems of scale insects was a recent review by Gavrilov (2007), which lists published information on genetic systems in scale insects. For information on endosymbiont status and identity, the primary source was Buchner's (1965) extensive monograph on endosymbiosis and the references therein. Another important source was ScaleNet, an online database that collects an extensive amount of data on scale insect biology and is also an important source for literature on scale insects. In addition, new references were identified via Web of Science and Google Scholar as well as by inspecting the references of all papers of interest and by searching for citations of key papers. The taxonomy used in the analysis was based on the arrangement of families given in ScaleNet (Ben-Dov et al. 2011), which reflects recent research and expert opinion (Hodgson and Foldi 2006; Gullan and Cook 2007). Figure 1 provides a schematic of the relationships between the scale insect families for which data were available in this analysis. For a few families, no data were available for any of the considered factors. These are the Carayonemidae, Coelostomidiidae, Pityococcidae and Stigmacoccidae (Archaeococcoidea), and the Micrococcidae (Neococcoidea). In species of the family Stictococcidae, only females harbor endosymbionts. Following the rationale of Normark—that it is only the presence of endosymbionts in males that selects for male killing— we included the Stictococcidae as “endosymbionts absent.”

### Analysis

Phylogenetic inertia can cause statistical problems for comparative analyses, as closely related taxa are more similar to one another than more distantly related taxa are, thus violating the assumption of statistical independence (Felsenstein 1985). Therefore, in order to obtain reliable estimates from a comparative analysis, it is important to include information on the evolutionary relationships between the taxa included in the analysis. This information can come from taxonomy or from phylogenetic inferences (Harvey and Pagel 1991).

Several molecular phylogenies describing the relationships between scale insects have recently been published. Some of these studies focus on relationship between species within the different families—Diaspididae (Morse and Normark 2006), Planococcidae (Downie and Gullan 2004; Hardy et al. 2008), Eriococcidae (Cook and Gullan 2004), and Monophlebiae (Unruh and Gullan 2008). Other studies explore the relationships between families (Cook et al. 2002; Gullan and Cook 2007). Although sequence data are available for more than 250 species, many of the relationships remain poorly resolved, especially at higher taxonomic levels.

The method adopted in this paper is to use a generalized linear mixed model (GLMM) approach where the relationship between taxa can be fitted as a random effect. For a taxonomic GLMM, taxonomic classification (for example, order, family, genus) can be fitted to include species relationships while in a phylogenetic GLMM the phylogenetic relationship between species can be fitted as a random effect (based on branch lengths between nodes) (Hadfield and Nakagawa 2010). The latter would be the preferred method, but reliable phylogenies are not always available. We chose not to use a species-level phylogeny partly because of the issues described above and partly because although sequence data are available for a reasonable number of scale insect species (roughly 500 species), there is little overlap with the species for which relevant data on genetic systems and endo-symbionts are available. We included family as a random effect, but chose not to include genus, as there is little variation in genetic system or endosymbiont status within families (see Results). As such, our analysis assumes a largely polytomic relationship between families. However, in order to include some information on the higher relationships between families, we divided the families into two groups, the Archaeococcoidea and the Neococcoidea. Although the Archaeococcoidea is almost certainly paraphyletic, the Neococcoidea is one of the few well-supported supra-family groupings within scale insects (Gullan and Cook 2007) (Fig. 1).

**Figure 2:**
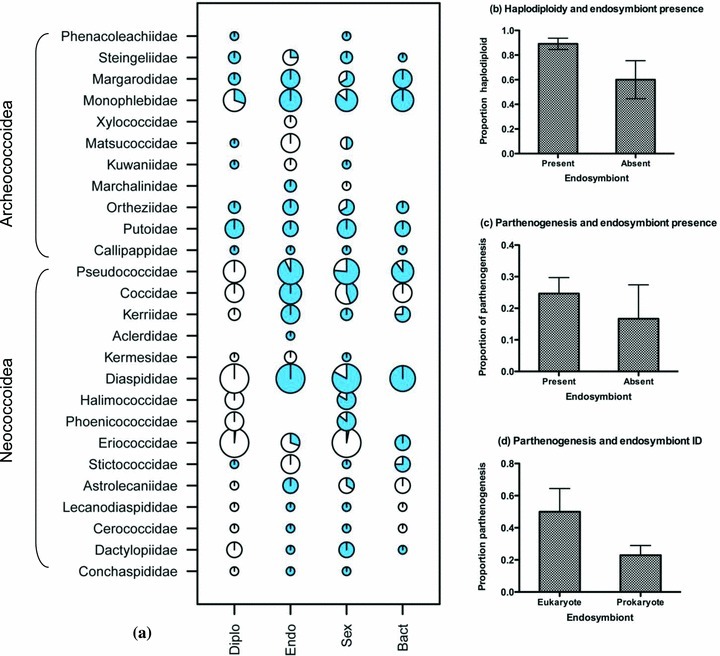
(a) Summary of the data used for the analysis. Pie-charts show the proportion per family for each of the four characters: proportion of species with diplodiploidy (Diplo, with diplodiploidy present in turquoise), proportion of species that reproduce sexually (Sex, with sexual in turquoise), endosymbiont presence (Endo, with species containing endosymbionts in turquoise), and proportion of endosymbionts that are prokaryote (Bact, with species containing bacterial endosymbionts in turquoise). Size of the pie reflects sample size for each family. (b) Proportion of species with haplodiploidy for species with and without endosymbionts. Error bars show the binomial standard errors. The graph only includes species for which data were available for both characters. (c) Proportion of species with asexual reproduction for species with and without endosymbionts. Error bars show the binomial standard errors. (d) Proportion of species with asexual reproduction for species with prokaryote and eukaryote endosymbionts. Error bars show the binomial standard errors.

We tested for a relationship between our characters of interest—genetic system, reproductive mode (sexual vs. asexual reproduction), endosymbiont presence and identity, and bacteriome presence (see Fig. 2)—using a bivariate binary mixed model approach. The models were fitted using the R package MCMCglmm (Hadfield 2010a), which provides a Bayesian framework for generalized mixed model analysis. We used a multivariate normal prior for the fixed effects with a null mean vector and a diagonal covariance matrix with variances of 10+π^2^/3, which is approximately flat on the probability scale when the sum of the variance components is 10 (roughly the posterior mode). An inverse Wishart prior was used for the family covariance matrix, with the covariance matrix at the limit set to an identity matrix, and degree of belief parameter 1.002. This is equivalent to having marginal inverse gamma priors for each variance, with the scale and shape parameters set to 0.001 (Spiegelhalter et al. 2003). We also used a prior with the covariance matrix at the limit set to a diagonal matrix with the variances set to 10+π^2^/3, and degree of belief parameter set to 3. This is an approximately flat prior for the correlation coefficient (see also Cornwallis et al. 2010 for an example of this approach; Hadfield 2010b; J. D. Hadfield, pers. comm.). The residual variances were fixed at one because they are not identifiable in binary models, and the residual correlation was set to zero. We compared the model outcomes using these different priors as well as varying the degree of belief parameter and the resulting models gave qualitatively (and quantitatively) similar answers. The residual correlation is generally estimable in bivariate binary models, but in each of the different analyses performed very few families were variable for both traits considered, thus there is little information to estimate the within-family correlation. An additional model in which the residual correlation was estimated (with a weak prior; Barnard et al. (2000) prior with degree of belief parameter equal to 5) was run for each analysis and gave qualitatively (and quantitatively) similar answers. The models were all run for 13 million iterations and the first 3 million iterations were discarded to ensure that the models had converged.

The analysis calculates the correlation coefficient between two response variables on the link scale at the family level. For simplicity, we will denote this correlation coefficient as *r*. For each estimate, we also calculate the 95% credibility interval (a Bayesian analogue to the confidence interval), which we will refer to as a 95% CI. We consider the correlation between two factors to be statistically significant if the 95% credibility estimates do not include zero. In order to test how much of the variation of a given response is explained by “family,” we calculated the intraclass correlation (correlation between the estimated phenotype of two species within the same family) given by Hadfield (2010a), as a measure of phylogenetic signal:

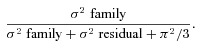


In order to test if taxonomic group (Archaeococcoidea vs. Neococcoidea) had a significant effect on the different factors considered in this analysis, we estimated the fixed effects (taxonomic group was fitted as a fixed effect, as it only has two levels), presented as the posterior mode and 95% CI.

## Results

The presence of diplodiplody was strongly associated with the absence of endosymbionts *r*=–0.96 (95% CI –1.00 to –0.61), *n*= 432 (Fig. 2b). Family explained most of the variation for both the presence of endosymbionts and that of diplodiploidy. The intraclass correlation was *r*= 0.72 (95% CI 0.36–0.95) for the presence of diplodiploidy and *r*= 0.97 (95% CI 0.83–0.99) for presence of endosymbionts. There was also a significant difference between the Neococcoids and Archaeococcoids in the presence of diplodiploidy (fraction of species with diplodiploidy, Archaeococcoidea = 0.60, Neococcoidea = 0.008, posterior mode: –8.89, 95% CI –12.72 to –5.74). However, there was no significant difference between the two scale insect taxonomic groups in terms of the occurrence of endosymbionts (fraction of species with endosymbionts, Archaeococcoidea = 0.76, Neococcoidea = 0.89, posterior mode: 0.36, 95% CI =–3.91 to 5.96).

We also tested for a possible relationship between endosymbiont presence and reproductive mode (asexual vs. sexual reproduction). First of all, there was much more within-family variation in reproductive mode than in the occurrence of diplodiploidy (the intraclass correlation for reproductive mode: *r =* 0.35, 95% CI 0.10–0.55). There was a negative correlation between endosymbiont presence and reproductive mode, however the effect is not significant (*r*=–0.89, 95% CI –0.98 to 0.17, *n*= 475; Fig. 2c). This suggests that endosymbiont presence does not significantly correlate with the presence of asexual reproduction. We also tested if the reproductive mode (sexual vs. asexual reproduction) was related to the identity of the endosymbiont. We found that there was a correlation between reproductive mode and symbiont identity, with asexuality being more common in species that contain eukaryote endosymbionts and this relationship was significant (*r*=–0.95, 95% CI –0.99 to –0.23, *n*= 447; Fig. 2d).

Finally, we tested if the identity of endosymbionts was associated with how closely integrated the endosymbionts are by the host (i.e., in a specialized organ or not). We found that eukaryote endosymbionts were less likely to be housed within a bacteriome (*r*=–0.97, 95% CI –0.99 to –0.27). The intraclass correlation of both factors was high, suggesting that family explains a large part of the variation in these factors (endosymbiont identity: *r*= 0.86, 95% CI = 0.59–0.99; bacteriome: *r*= 0.85, 95% CI = 0.60–0.97, *n*= 238).

## Discussion

Scale insects that possess endosymbionts are more likely to have a haplodiploid genetic system. This supports the hypothesis postulated by Normark (2004) who considered that coevolution between host and endosymbionts with a male-killing phenotype could have led to the evolution of male-haploid genetic systems. The results presented here are the first formal analysis that shows support for this hypothesis. Male-haploid genetic systems are found in a large number of taxa of both insects and mites. It will be of great interest to see if a similar association with endosymbionts can be observed in these systems.

Our results are based on a correlation between haplodiploidy and endosymbiont presence, but Normark's (2004) hypothesis is also further supported by a direct observation that reversion from PGE to diplodiploidy has occurred in the scale insect family Stictococcidae and is associated with a lost of symbionts in the male sex (Buchner 1955, 1965; Ross et al. 2010). Females of this family still contain symbionts and the lack of the essential endosymbionts in males is compensated for by the evolution of a placenta-like structure, with which the mother directly feeds her offspring. This is one out of the only two cases of reversal from haplodiploidy that have been recorded (Normark 2004); the other case is also found in scale insects (the genus *Lachnodius*), but unfortunately here the endosymbiont status is unknown. Another interesting recent observation comes from a parasitoid wasp in which the absence of their usual parthenogenesis-inducing bacteria leads to the development of diploid males (Giorgini et al. 2009).

A general problem with the type of comparative analysis preformed here is that it considers correlations between traits but cannot make any inference about the direction of causality. It is therefore in principle a possibility that our results can be explained by the alternative hypothesis that haplodiploidy promotes the acquisition of endosymbionts. There is, however, no theoretical support for this alternative. Yet another possible hypothesis would be that haplodiploidy and endosymbiont acquisition are both promoted by some other common cause (environment, food source, etc.). Two such factors could be inbreeding and poor diet, as endo-symbiont acquisition has been suggested to correlate with the latter, while haplodiploidy seems to correlate with the former (Smith 2000). As pointed out by Normark (2004), five to seven of the 10 haplodiploid insect clades primitively feed within cavities in dead woody tissue, a lifestyle that could both promote the acquisition of endosymbionts as well as strong interactions between kin (including sibmating). So, if inbreeding by itself can explain the evolution of haplodiploidy, this might serve as an alternative for the pattern we observe. The theoretical support for the driving role of inbreeding in the evolution of haplodiploidy, however, is ambiguous (Smith 2000), and inbreeding may in fact limit the evolution of haplodiploidy (Smith 2000). On the other hand, the likelihood of endosymbiont-induced transitions to haplodiploidy is more likely under strong inbreeding (Kuijper and Pen 2010).

Normark's (2004) hypothesis considers coevolution between male-killing endosymbionts and their hosts as the driving force behind the transition to male-haploid genetic systems. However, recently it has been shown that another phenotype commonly induced by endosymbionts, cytoplasmic incompatibility (CI), might also lead to conflict between host and symbiont resulting in the evolution of male-haploid genetic systems (Engelstadter and Hurst 2006). Both mechanisms are consistent with our findings here.

Although we find a relationship between genetic system and endosymbiont presence, we do not find a relationship with reproductive mode (i.e., sexual vs. asexual). But the type of endosymbiont (bacteria vs. unicellular fungi) does correlate with reproductive mode, with asexual reproduction more often found in species with eukaryote endosymbionts. This could be because the latter are generally distributed freely in the hemolymph and are even found to be able to penetrate a variety of cells (including germline cells) (Tremblay 1989). This means that they might be less tightly controlled by their host than bacterial endosymbionts, which are generally restricted to specialized cells, giving them more opportunity to influence their host's reproduction (Ross et al. 2010).

The role of endosymbionts in the evolution of asexual reproduction has been well established in many insects (Hurst et al. 1990; Stouthamer et al. 1990; Werren 1997; Weeks et al. 2002, 2003; Koivisto and Braig 2003; Zchori-Fein and Perlman 2004; Duron et al. 2008; Werren et al. 2008) and the endosymbiotic bacterium *Cardinium* is associated with parthenogenesis in a species of armored scale insect (Provencher et al. 2005). The fact that in our analysis we do not find strong support for a generalized role of endosymbionts on the presence of asexual reproduction in scale insects might be due to the fact that in our analysis we simply used presence or absence, and were unable to distinguish between primary and secondary symbionts, as the role of the symbionts described in scale insects are mostly unknown. However, previous analyses have mainly found that the effect of secondary (often purely parasitic) bacteria is associated with asexual reproduction (both in scale insects, Gruwell et al. 2009; Nur 1972; as well as in other taxa Werren et al. 2008). More work on scale insect endosymbionts is therefore clearly merited.

Apart from the presence of endosymbionts, population structures leading to high levels of kin competition is an important assumption of Normark's model, as selection on the endosymbiont for a male-killing phenotype will only be strong under such conditions. Scale insects usually have gregarious clutches and have evolved a variety of ways in which eggs and larvae are protected by the mother (in a marsupium, or ovisac for example). Additionally, crawlers often settle close to their mothers (Gullan and Kosztarab 1997). All these factors lead to prolonged associations between kin and could therefore lead to high levels of kin competition (Normark 2004, 2006; Ross et al. 2010). This might help to explain how endosymbiosis in scale insects could have led to conflict between endosymbiont and host, and resulted in a change in the host's genetic system. Reduced sib competition might help to explain the few cases where endosymbiosis has not resulted in the evolution of haplodiploidy, although sib competition is hard to quantify. (Data might be available on factors that could correlate with the level of sib competition, for example, the presence of gregarious clutches, the clutch size, and how mothers protect their eggs.) Such data might also in the future be able to distinguish between the importance of male killing vs. CI, as only male-killing requires sib competition.

Recently the importance of genomic conflict in shaping defining characteristics of genomic organization and key aspects of biology such as reproductive mode and genetic system has become apparent and received considerable attention (extensively reviewed in Burt and Trivers 2006). However, few studies have actually made an attempt to test these hypotheses formally. Further comparative analyses such as the one undertaken here will help to increase our understanding of the evolutionary importance of genetic conflict.

## Supporting Information - Evolutionary Analysis

**Table S1.** List of species and references for the data that were included in the analysis.
